# Supplementary material for: Chromothripsis during telomere crisis is independent of NHEJ, and consistent with a replicative origin
Source: Genome Res. 2019 May;29(5):737–49. doi: 10.1101/gr.240705.118 (PMC6499312; doi:10.1101/gr.240705.118)
Supplement: Supplemental Material [file supp_gr.240705.118_Supplemental_file_1.zip › contigs/annotated_contigs/DB79/contig.2.DB79_length_449_mean_cov_6.34743875278.docx]

**DB79_length_449_mean_cov_6.34743875278**

```
TGGCATGAAGGCCTCTGGTGATCAGATTAGCCCTCTGTGACTTTCCCCTCCCCTGGGTTCCCATAAGAAATGGCTGAACCACTCCTTTG
 >chr1:180126944-180127125 - E=2e-94
GCCTTTTTCAAACACAGCTTTGTAACAGTTGCCAATTTTCCTTCTATGAGTAGCTCATTTCCCCAAATGCAATGCAAACAATGCTTA|T

CACT|GGATAATTTACTTGTTGCAGCATCAAACCCAGCAGTGGCCTATGGACAAAGCGCAGAGCCAGGGGCTTACACAGGGGACAGAAA
>chr1:180220495-180220768 - E=8e-147
AATGAGTAACAGATAGGCCTTTTCCTCAAGCCAACAAGGAAGATAAACCAGGCACACGAGAAATGGGCAAACCAAAGCATTNTGTGCTG

GGTCACCCGGGAGAGGGGAGAGACAGGAAGTGATGGCTCCACTGGGGGTCCTGTCTGCCGCGGAGCTGGACTCTGAGGACCCTGGAAGG

ATGGAA
```
